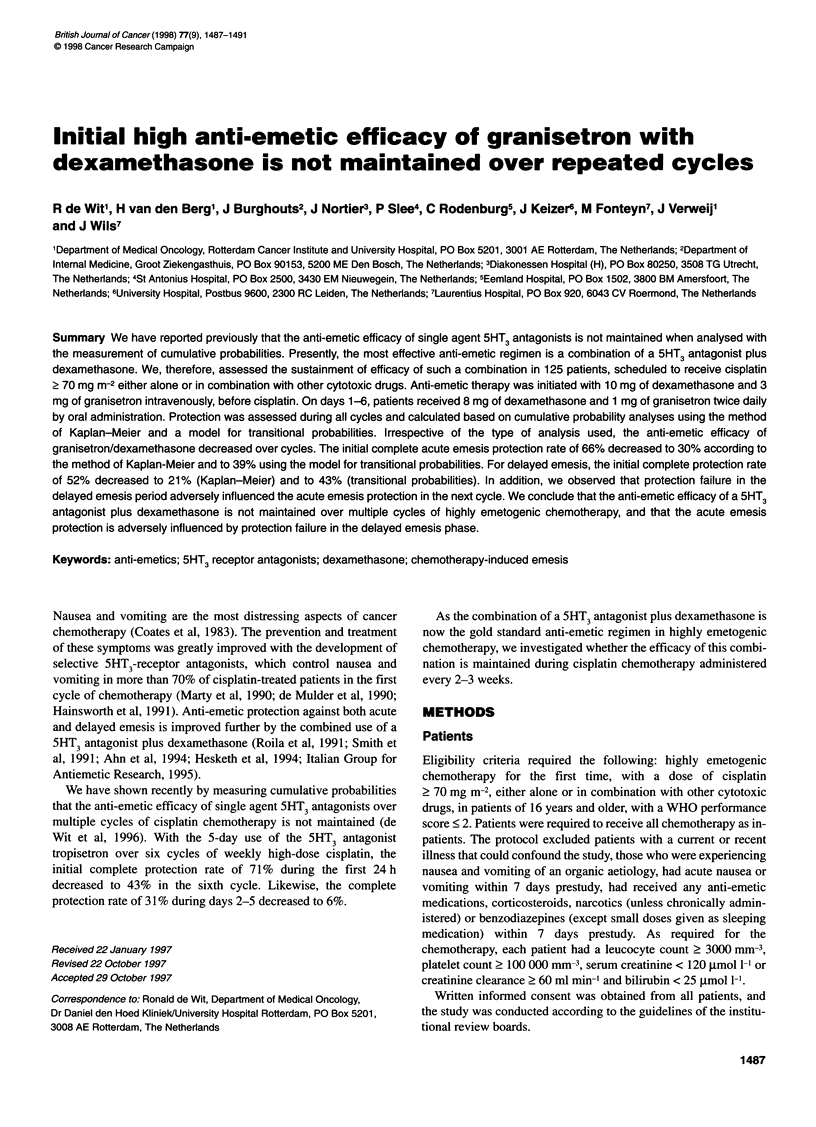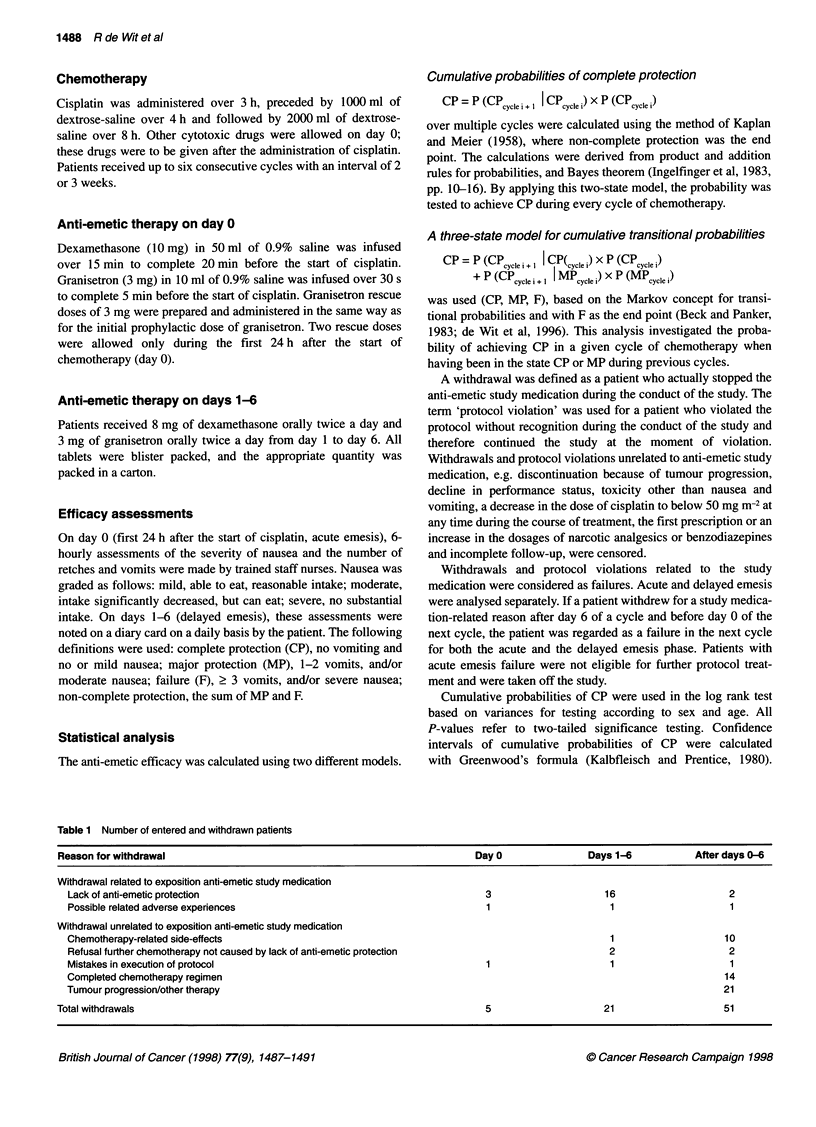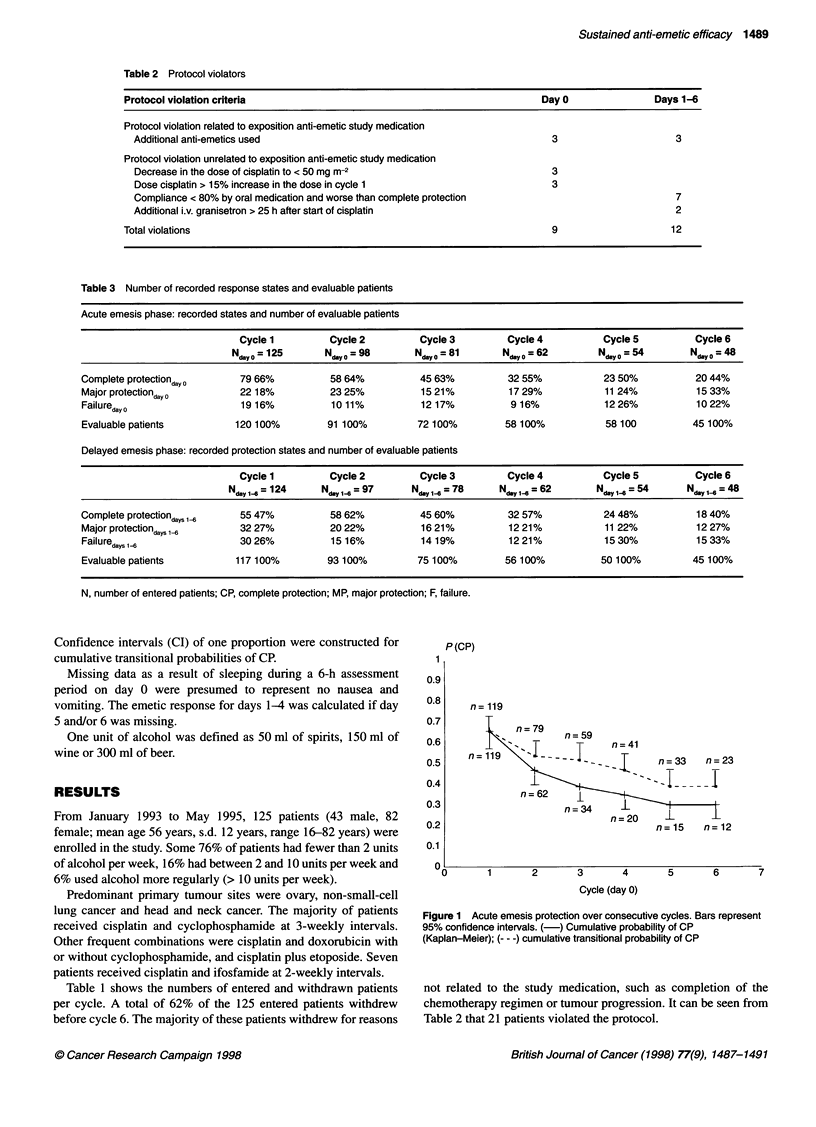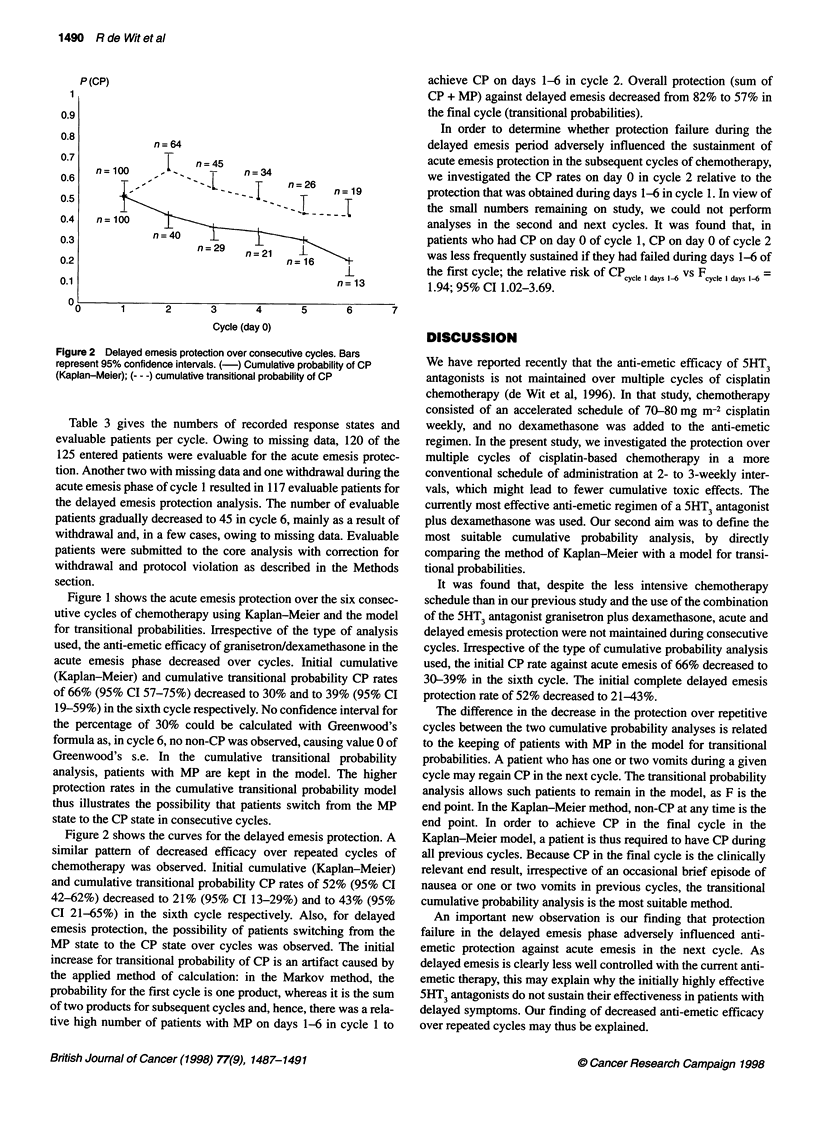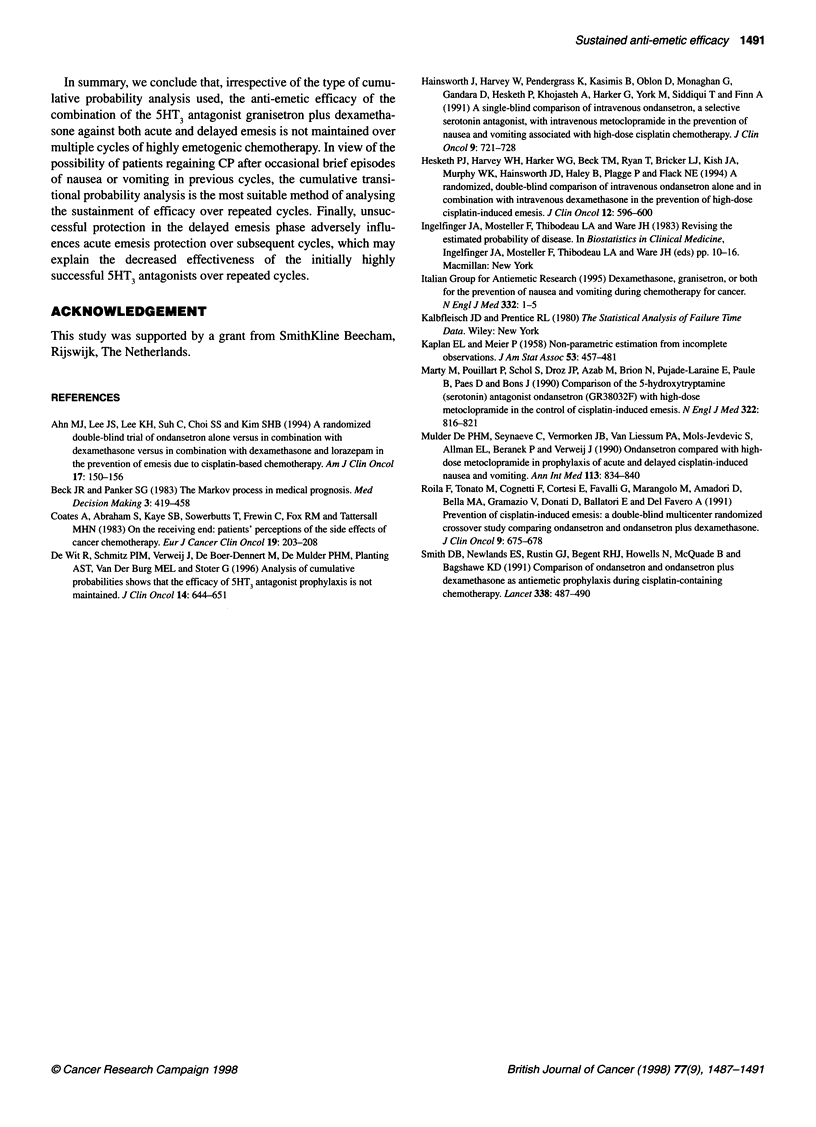# Initial high anti-emetic efficacy of granisetron with dexamethasone is not maintained over repeated cycles.

**DOI:** 10.1038/bjc.1998.244

**Published:** 1998-05

**Authors:** R. de Wit, H. van den Berg, J. Burghouts, J. Nortier, P. Slee, C. Rodenburg, J. Keizer, M. Fonteyn, J. Verweij, J. Wils

**Affiliations:** Department of Medical Oncology, Rotterdam Cancer Institute and University Hospital, The Netherlands.

## Abstract

We have reported previously that the anti-emetic efficacy of single agent 5HT3 antagonists is not maintained when analysed with the measurement of cumulative probabilities. Presently, the most effective anti-emetic regimen is a combination of a 5HT3 antagonist plus dexamethasone. We, therefore, assessed the sustainment of efficacy of such a combination in 125 patients, scheduled to receive cisplatin > or = 70 mg m(-2) either alone or in combination with other cytotoxic drugs. Anti-emetic therapy was initiated with 10 mg of dexamethasone and 3 mg of granisetron intravenously, before cisplatin. On days 1-6, patients received 8 mg of dexamethasone and 1 mg of granisetron twice daily by oral administration. Protection was assessed during all cycles and calculated based on cumulative probability analyses using the method of Kaplan-Meier and a model for transitional probabilities. Irrespective of the type of analysis used, the anti-emetic efficacy of granisetron/dexamethasone decreased over cycles. The initial complete acute emesis protection rate of 66% decreased to 30% according to the method of Kaplan-Meier and to 39% using the model for transitional probabilities. For delayed emesis, the initial complete protection rate of 52% decreased to 21% (Kaplan-Meier) and to 43% (transitional probabilities). In addition, we observed that protection failure in the delayed emesis period adversely influenced the acute emesis protection in the next cycle. We conclude that the anti-emetic efficacy of a 5HT3 antagonist plus dexamethasone is not maintained over multiple cycles of highly emetogenic chemotherapy, and that the acute emesis protection is adversely influenced by protection failure in the delayed emesis phase.


British Journal of Cancer (1998) 77(9), 1487–1491
© 1998 Cancer Research Campaign

### Initial high anti-emetic efficacy of granisetron with dexamethasone is not maintained over repeated cycles

**R de Wit[1], H van den Berg[1], J Burghouts[2], J Nortier[3], P Slee[4], C Rodenburg[5], J Keizer[6], M Fonteyn[7], J Verweij[1] and J Wils[7]**

[1]Department of Medical Oncology, Rotterdam Cancer Institute and University Hospital, PO Box 5201, 3001 AE Rotterdam, The Netherlands; [2]Department of Internal Medicine, Groot Ziekengasthuis, PO Box 90153, 5200 ME Den Bosch, The Netherlands; [3]Diakonessen Hospital (H), PO Box 80250, 3508 TG Utrecht, The Netherlands; [4]St Antonius Hospital, PO Box 2500, 3430 EM Nieuwegein, The Netherlands; [5]Eemland Hospital, PO Box 1502, 3800 BM Amersfoort, The Netherlands; [6]University Hospital, Postbus 9600, 2300 RC Leiden, The Netherlands; [7]Laurentius Hospital, PO Box 920, 6043 CV Roermond, The Netherlands

**Summary** We have reported previously that the anti-emetic efficacy of single agent $5HT_3$ antagonists is not maintained when analysed with the measurement of cumulative probabilities. Presently, the most effective anti-emetic regimen is a combination of a $5HT_3$ antagonist plus dexamethasone. We, therefore, assessed the sustainment of efficacy of such a combination in 125 patients, scheduled to receive cisplatin $\geq$ 70 mg m$^{-2}$ either alone or in combination with other cytotoxic drugs. Anti-emetic therapy was initiated with 10 mg of dexamethasone and 3 mg of granisetron intravenously, before cisplatin. On days 1–6, patients received 8 mg of dexamethasone and 1 mg of granisetron twice daily by oral administration. Protection was assessed during all cycles and calculated based on cumulative probability analyses using the method of Kaplan–Meier and a model for transitional probabilities. Irrespective of the type of analysis used, the anti-emetic efficacy of granisetron/dexamethasone decreased over cycles. The initial complete acute emesis protection rate of 66% decreased to 30% according to the method of Kaplan-Meier and to 39% using the model for transitional probabilities. For delayed emesis, the initial complete protection rate of 52% decreased to 21% (Kaplan–Meier) and to 43% (transitional probabilities). In addition, we observed that protection failure in the delayed emesis period adversely influenced the acute emesis protection in the next cycle. We conclude that the anti-emetic efficacy of a $5HT_3$ antagonist plus dexamethasone is not maintained over multiple cycles of highly emetogenic chemotherapy, and that the acute emesis protection is adversely influenced by protection failure in the delayed emesis phase.

**Keywords:** anti-emetics; $5HT_3$ receptor antagonists; dexamethasone; chemotherapy-induced emesis

Nausea and vomiting are the most distressing aspects of cancer chemotherapy (Coates et al, 1983). The prevention and treatment of these symptoms was greatly improved with the development of selective $5HT_3$-receptor antagonists, which control nausea and vomiting in more than 70% of cisplatin-treated patients in the first cycle of chemotherapy (Marty et al, 1990; de Mulder et al, 1990; Hainsworth et al, 1991). Anti-emetic protection against both acute and delayed emesis is improved further by the combined use of a $5HT_3$ antagonist plus dexamethasone (Roila et al, 1991; Smith et al, 1991; Ahn et al, 1994; Hesketh et al, 1994; Italian Group for Antiemetic Research, 1995).

We have shown recently by measuring cumulative probabilities that the anti-emetic efficacy of single agent $5HT_3$ antagonists over multiple cycles of cisplatin chemotherapy is not maintained (de Wit et al, 1996). With the 5-day use of the $5HT_3$ antagonist tropisetron over six cycles of weekly high-dose cisplatin, the initial complete protection rate of 71% during the first 24 h decreased to 43% in the sixth cycle. Likewise, the complete protection rate of 31% during days 2–5 decreased to 6%.

*Received 22 January 1997*
*Revised 22 October 1997*
*Accepted 29 October 1997*

*Correspondence to:* Ronald de Wit, Department of Medical Oncology, Dr Daniel den Hoed Kliniek/University Hospital Rotterdam, PO Box 5201, 3008 AE Rotterdam, The Netherlands

As the combination of a $5HT_3$ antagonist plus dexamethasone is now the gold standard anti-emetic regimen in highly emetogenic chemotherapy, we investigated whether the efficacy of this combination is maintained during cisplatin chemotherapy administered every 2–3 weeks.

### METHODS

### Patients

Eligibility criteria required the following: highly emetogenic chemotherapy for the first time, with a dose of cisplatin $\geq$ 70 mg m$^{-2}$, either alone or in combination with other cytotoxic drugs, in patients of 16 years and older, with a WHO performance score $\leq$ 2. Patients were required to receive all chemotherapy as in-patients. The protocol excluded patients with a current or recent illness that could confound the study, those who were experiencing nausea and vomiting of an organic aetiology, had acute nausea or vomiting within 7 days prestudy, had received any anti-emetic medications, corticosteroids, narcotics (unless chronically administered) or benzodiazepines (except small doses given as sleeping medication) within 7 days prestudy. As required for the chemotherapy, each patient had a leucocyte count $\geq$ 3000 mm$^{-3}$, platelet count $\geq$ 100 000 mm$^{-3}$, serum creatinine < 120 μmol l$^{-1}$ or creatinine clearance $\geq$ 60 ml min$^{-1}$ and bilirubin < 25 μmol l$^{-1}$.

Written informed consent was obtained from all patients, and the study was conducted according to the guidelines of the institutional review boards.

### Chemotherapy

Cisplatin was administered over 3 h, preceded by 1000 ml of dextrose-saline over 4 h and followed by 2000 ml of dextrose-saline over 8 h. Other cytotoxic drugs were allowed on day 0; these drugs were to be given after the administration of cisplatin. Patients received up to six consecutive cycles with an interval of 2 or 3 weeks.

### Anti-emetic therapy on day 0

Dexamethasone (10 mg) in 50 ml of 0.9% saline was infused over 15 min to complete 20 min before the start of cisplatin. Granisetron (3 mg) in 10 ml of 0.9% saline was infused over 30 s to complete 5 min before the start of cisplatin. Granisetron rescue doses of 3 mg were prepared and administered in the same way as for the initial prophylactic dose of granisetron. Two rescue doses were allowed only during the first 24 h after the start of chemotherapy (day 0).

### Anti-emetic therapy on days 1–6

Patients received 8 mg of dexamethasone orally twice a day and 3 mg of granisetron orally twice a day from day 1 to day 6. All tablets were blister packed, and the appropriate quantity was packed in a carton.

### Efficacy assessments

On day 0 (first 24 h after the start of cisplatin, acute emesis), 6-hourly assessments of the severity of nausea and the number of retches and vomits were made by trained staff nurses. Nausea was graded as follows: mild, able to eat, reasonable intake; moderate, intake significantly decreased, but can eat; severe, no substantial intake. On days 1–6 (delayed emesis), these assessments were noted on a diary card on a daily basis by the patient. The following definitions were used: complete protection (CP), no vomiting and no or mild nausea; major protection (MP), 1–2 vomits, and/or moderate nausea; failure (F), ≥ 3 vomits, and/or severe nausea; non-complete protection, the sum of MP and F.

### Statistical analysis

The anti-emetic efficacy was calculated using two different models.

### *Cumulative probabilities of complete protection*

$$CP = P(CP_{cycle\ i+1} \mid CP_{cycle\ i}) \times P(CP_{cycle\ i})$$

over multiple cycles were calculated using the method of Kaplan and Meier (1958), where non-complete protection was the end point. The calculations were derived from product and addition rules for probabilities, and Bayes theorem (Ingelfinger et al, 1983, pp. 10–16). By applying this two-state model, the probability was tested to achieve CP during every cycle of chemotherapy.

### *A three-state model for cumulative transitional probabilities*

$$CP = P(CP_{cycle\ i+1} \mid CP(_{cycle\ i}) \times P(CP_{cycle\ i}) + P(CP_{cycle\ i+1} \mid MP_{cycle\ i}) \times P(MP_{cycle\ i})$$

was used (CP, MP, F), based on the Markov concept for transitional probabilities and with F as the end point (Beck and Panker, 1983; de Wit et al, 1996). This analysis investigated the probability of achieving CP in a given cycle of chemotherapy when having been in the state CP or MP during previous cycles.

A withdrawal was defined as a patient who actually stopped the anti-emetic study medication during the conduct of the study. The term 'protocol violation' was used for a patient who violated the protocol without recognition during the conduct of the study and therefore continued the study at the moment of violation. Withdrawals and protocol violations unrelated to anti-emetic study medication, e.g. discontinuation because of tumour progression, decline in performance status, toxicity other than nausea and vomiting, a decrease in the dose of cisplatin to below 50 mg $m^{-2}$ at any time during the course of treatment, the first prescription or an increase in the dosages of narcotic analgesics or benzodiazepines and incomplete follow-up, were censored.

Withdrawals and protocol violations related to the study medication were considered as failures. Acute and delayed emesis were analysed separately. If a patient withdrew for a study medication-related reason after day 6 of a cycle and before day 0 of the next cycle, the patient was regarded as a failure in the next cycle for both the acute and the delayed emesis phase. Patients with acute emesis failure were not eligible for further protocol treatment and were taken off the study.

Cumulative probabilities of CP were used in the log rank test based on variances for testing according to sex and age. All *P*-values refer to two-tailed significance testing. Confidence intervals of cumulative probabilities of CP were calculated with Greenwood's formula (Kalbfleisch and Prentice, 1980).

**Table 1** Number of entered and withdrawn patients

| Reason for withdrawal | Day 0 | Days 1–6 | After days 0–6 |
|---|---|---|---|
| Withdrawal related to exposition anti-emetic study medication | | | |
| Lack of anti-emetic protection | 3 | 16 | 2 |
| Possible related adverse experiences | 1 | 1 | 1 |
| Withdrawal unrelated to exposition anti-emetic study medication | | | |
| Chemotherapy-related side-effects | | 1 | 10 |
| Refusal further chemotherapy not caused by lack of anti-emetic protection | | 2 | 2 |
| Mistakes in execution of protocol | 1 | 1 | 1 |
| Completed chemotherapy regimen | | | 14 |
| Tumour progression/other therapy | | | 21 |
| Total withdrawals | 5 | 21 | 51 |

© Cancer Research Campaign 1998

**Table 2** Protocol violators

| Protocol violation criteria | Day 0 | Days 1–6 |
|---|---|---|
| Protocol violation related to exposition anti-emetic study medication | | |
| Additional anti-emetics used | 3 | 3 |
| Protocol violation unrelated to exposition anti-emetic study medication | | |
| Decrease in the dose of cisplatin to < 50 mg $m^{-2}$ | 3 | |
| Dose cisplatin > 15% increase in the dose in cycle 1 | 3 | |
| Compliance < 80% by oral medication and worse than complete protection | | 7 |
| Additional i.v. granisetron > 25 h after start of cisplatin | | 2 |
| Total violations | 9 | 12 |

**Table 3** Number of recorded response states and evaluable patients

Acute emesis phase: recorded states and number of evaluable patients

| | Cycle 1 $N_{day\,0} = 125$ | Cycle 2 $N_{day\,0} = 98$ | Cycle 3 $N_{day\,0} = 81$ | Cycle 4 $N_{day\,0} = 62$ | Cycle 5 $N_{day\,0} = 54$ | Cycle 6 $N_{day\,0} = 48$ |
|---|---|---|---|---|---|---|
| Complete protection$_{day\,0}$ | 79 66% | 58 64% | 45 63% | 32 55% | 23 50% | 20 44% |
| Major protection$_{day\,0}$ | 22 18% | 23 25% | 15 21% | 17 29% | 11 24% | 15 33% |
| Failure$_{day\,0}$ | 19 16% | 10 11% | 12 17% | 9 16% | 12 26% | 10 22% |
| Evaluable patients | 120 100% | 91 100% | 72 100% | 58 100% | 58 100 | 45 100% |

Delayed emesis phase: recorded protection states and number of evaluable patients

| | Cycle 1 $N_{day\,1\text{–}6} = 124$ | Cycle 2 $N_{day\,1\text{–}6} = 97$ | Cycle 3 $N_{day\,1\text{–}6} = 78$ | Cycle 4 $N_{day\,1\text{–}6} = 62$ | Cycle 5 $N_{day\,1\text{–}6} = 54$ | Cycle 6 $N_{day\,1\text{–}6} = 48$ |
|---|---|---|---|---|---|---|
| Complete protection$_{days\,1\text{–}6}$ | 55 47% | 58 62% | 45 60% | 32 57% | 24 48% | 18 40% |
| Major protection$_{days\,1\text{–}6}$ | 32 27% | 20 22% | 16 21% | 12 21% | 11 22% | 12 27% |
| Failure$_{days\,1\text{–}6}$ | 30 26% | 15 16% | 14 19% | 12 21% | 15 30% | 15 33% |
| Evaluable patients | 117 100% | 93 100% | 75 100% | 56 100% | 50 100% | 45 100% |

N, number of entered patients; CP, complete protection; MP, major protection; F, failure.

Confidence intervals (CI) of one proportion were constructed for cumulative transitional probabilities of CP.

Missing data as a result of sleeping during a 6-h assessment period on day 0 were presumed to represent no nausea and vomiting. The emetic response for days 1–4 was calculated if day 5 and/or 6 was missing.

One unit of alcohol was defined as 50 ml of spirits, 150 ml of wine or 300 ml of beer.

### RESULTS

From January 1993 to May 1995, 125 patients (43 male, 82 female; mean age 56 years, s.d. 12 years, range 16–82 years) were enrolled in the study. Some 76% of patients had fewer than 2 units of alcohol per week, 16% had between 2 and 10 units per week and 6% used alcohol more regularly (> 10 units per week).

Predominant primary tumour sites were ovary, non-small-cell lung cancer and head and neck cancer. The majority of patients received cisplatin and cyclophosphamide at 3-weekly intervals. Other frequent combinations were cisplatin and doxorubicin with or without cyclophosphamide, and cisplatin plus etoposide. Seven patients received cisplatin and ifosfamide at 2-weekly intervals.

Table 1 shows the numbers of entered and withdrawn patients per cycle. A total of 62% of the 125 entered patients withdrew before cycle 6. The majority of these patients withdrew for reasons not related to the study medication, such as completion of the chemotherapy regimen or tumour progression. It can be seen from Table 2 that 21 patients violated the protocol.

**Figure 1** Acute emesis protection over consecutive cycles. Bars represent 95% confidence intervals. (——) Cumulative probability of CP (Kaplan–Meier); (- - -) cumulative transitional probability of CP

**Figure 2** Delayed emesis protection over consecutive cycles. Bars represent 95% confidence intervals. (——) Cumulative probability of CP (Kaplan–Meier); (- - -) cumulative transitional probability of CP

Table 3 gives the numbers of recorded response states and evaluable patients per cycle. Owing to missing data, 120 of the 125 entered patients were evaluable for the acute emesis protection. Another two with missing data and one withdrawal during the acute emesis phase of cycle 1 resulted in 117 evaluable patients for the delayed emesis protection analysis. The number of evaluable patients gradually decreased to 45 in cycle 6, mainly as a result of withdrawal and, in a few cases, owing to missing data. Evaluable patients were submitted to the core analysis with correction for withdrawal and protocol violation as described in the Methods section.

Figure 1 shows the acute emesis protection over the six consecutive cycles of chemotherapy using Kaplan–Meier and the model for transitional probabilities. Irrespective of the type of analysis used, the anti-emetic efficacy of granisetron/dexamethasone in the acute emesis phase decreased over cycles. Initial cumulative (Kaplan–Meier) and cumulative transitional probability CP rates of 66% (95% CI 57–75%) decreased to 30% and to 39% (95% CI 19–59%) in the sixth cycle respectively. No confidence interval for the percentage of 30% could be calculated with Greenwood's formula as, in cycle 6, no non-CP was observed, causing value 0 of Greenwood's s.e. In the cumulative transitional probability analysis, patients with MP are kept in the model. The higher protection rates in the cumulative transitional probability model thus illustrates the possibility that patients switch from the MP state to the CP state in consecutive cycles.

Figure 2 shows the curves for the delayed emesis protection. A similar pattern of decreased efficacy over repeated cycles of chemotherapy was observed. Initial cumulative (Kaplan–Meier) and cumulative transitional probability CP rates of 52% (95% CI 42–62%) decreased to 21% (95% CI 13–29%) and to 43% (95% CI 21–65%) in the sixth cycle respectively. Also, for delayed emesis protection, the possibility of patients switching from the MP state to the CP state over cycles was observed. The initial increase for transitional probability of CP is an artifact caused by the applied method of calculation: in the Markov method, the probability for the first cycle is one product, whereas it is the sum of two products for subsequent cycles and, hence, there was a relative high number of patients with MP on days 1–6 in cycle 1 to achieve CP on days 1–6 in cycle 2. Overall protection (sum of CP + MP) against delayed emesis decreased from 82% to 57% in the final cycle (transitional probabilities).

In order to determine whether protection failure during the delayed emesis period adversely influenced the sustainment of acute emesis protection in the subsequent cycles of chemotherapy, we investigated the CP rates on day 0 in cycle 2 relative to the protection that was obtained during days 1–6 in cycle 1. In view of the small numbers remaining on study, we could not perform analyses in the second and next cycles. It was found that, in patients who had CP on day 0 of cycle 1, CP on day 0 of cycle 2 was less frequently sustained if they had failed during days 1–6 of the first cycle; the relative risk of $CP_{\text{cycle 1 days 1–6}}$ vs $F_{\text{cycle 1 days 1–6}}$ = 1.94; 95% CI 1.02–3.69.

### DISCUSSION

We have reported recently that the anti-emetic efficacy of $5HT_3$ antagonists is not maintained over multiple cycles of cisplatin chemotherapy (de Wit et al, 1996). In that study, chemotherapy consisted of an accelerated schedule of 70–80 mg $m^{-2}$ cisplatin weekly, and no dexamethasone was added to the anti-emetic regimen. In the present study, we investigated the protection over multiple cycles of cisplatin-based chemotherapy in a more conventional schedule of administration at 2- to 3-weekly intervals, which might lead to fewer cumulative toxic effects. The currently most effective anti-emetic regimen of a $5HT_3$ antagonist plus dexamethasone was used. Our second aim was to define the most suitable cumulative probability analysis, by directly comparing the method of Kaplan–Meier with a model for transitional probabilities.

It was found that, despite the less intensive chemotherapy schedule than in our previous study and the use of the combination of the $5HT_3$ antagonist granisetron plus dexamethasone, acute and delayed emesis protection were not maintained during consecutive cycles. Irrespective of the type of cumulative probability analysis used, the initial CP rate against acute emesis of 66% decreased to 30–39% in the sixth cycle. The initial complete delayed emesis protection rate of 52% decreased to 21–43%.

The difference in the decrease in the protection over repetitive cycles between the two cumulative probability analyses is related to the keeping of patients with MP in the model for transitional probabilities. A patient who has one or two vomits during a given cycle may regain CP in the next cycle. The transitional probability analysis allows such patients to remain in the model, as F is the end point. In the Kaplan–Meier method, non-CP at any time is the end point. In order to achieve CP in the final cycle in the Kaplan–Meier model, a patient is thus required to have CP during all previous cycles. Because CP in the final cycle is the clinically relevant end result, irrespective of an occasional brief episode of nausea or one or two vomits in previous cycles, the transitional cumulative probability analysis is the most suitable method.

An important new observation is our finding that protection failure in the delayed emesis phase adversely influenced anti-emetic protection against acute emesis in the next cycle. As delayed emesis is clearly less well controlled with the current anti-emetic therapy, this may explain why the initially highly effective $5HT_3$ antagonists do not sustain their effectiveness in patients with delayed symptoms. Our finding of decreased anti-emetic efficacy over repeated cycles may thus be explained.

© Cancer Research Campaign 1998

In summary, we conclude that, irrespective of the type of cumulative probability analysis used, the anti-emetic efficacy of the combination of the $5HT_3$ antagonist granisetron plus dexamethasone against both acute and delayed emesis is not maintained over multiple cycles of highly emetogenic chemotherapy. In view of the possibility of patients regaining CP after occasional brief episodes of nausea or vomiting in previous cycles, the cumulative transitional probability analysis is the most suitable method of analysing the sustainment of efficacy over repeated cycles. Finally, unsuccessful protection in the delayed emesis phase adversely influences acute emesis protection over subsequent cycles, which may explain the decreased effectiveness of the initially highly successful $5HT_3$ antagonists over repeated cycles.

### ACKNOWLEDGEMENT

This study was supported by a grant from SmithKline Beecham, Rijswijk, The Netherlands.

### REFERENCES

Ahn MJ, Lee JS, Lee KH, Suh C, Choi SS and Kim SHB (1994) A randomized double-blind trial of ondansetron alone versus in combination with dexamethasone versus in combination with dexamethasone and lorazepam in the prevention of emesis due to cisplatin-based chemotherapy. *Am J Clin Oncol* **17**: 150–156

Beck JR and Panker SG (1983) The Markov process in medical prognosis. *Med Decision Making* **3**: 419–458

Coates A, Abraham S, Kaye SB, Sowerbutts T, Frewin C, Fox RM and Tattersall MHN (1983) On the receiving end: patients' perceptions of the side effects of cancer chemotherapy. *Eur J Cancer Clin Oncol* **19**: 203–208

De Wit R, Schmitz PIM, Verweij J, De Boer-Dennert M, De Mulder PHM, Planting AST, Van Der Burg MEL and Stoter G (1996) Analysis of cumulative probabilities shows that the efficacy of $5HT_3$ antagonist prophylaxis is not maintained. *J Clin Oncol* **14**: 644–651

Hainsworth J, Harvey W, Pendergrass K, Kasimis B, Oblon D, Monaghan G, Gandara D, Hesketh P, Khojasteh A, Harker G, York M, Siddiqui T and Finn A (1991) A single-blind comparison of intravenous ondansetron, a selective serotonin antagonist, with intravenous metoclopramide in the prevention of nausea and vomiting associated with high-dose cisplatin chemotherapy. *J Clin Oncol* **9**: 721–728

Hesketh PJ, Harvey WH, Harker WG, Beck TM, Ryan T, Bricker LJ, Kish JA, Murphy WK, Hainsworth JD, Haley B, Plagge P and Flack NE (1994) A randomized, double-blind comparison of intravenous ondansetron alone and in combination with intravenous dexamethasone in the prevention of high-dose cisplatin-induced emesis. *J Clin Oncol* **12**: 596–600

Ingelfinger JA, Mosteller F, Thibodeau LA and Ware JH (1983) Revising the estimated probability of disease. In *Biostatistics in Clinical Medicine*, Ingelfinger JA, Mosteller F, Thibodeau LA and Ware JH (eds) pp. 10–16. Macmillan: New York

Italian Group for Antiemetic Research (1995) Dexamethasone, granisetron, or both for the prevention of nausea and vomiting during chemotherapy for cancer. *N Engl J Med* **332**: 1–5

Kalbfleisch JD and Prentice RL (1980) *The Statistical Analysis of Failure Time Data*. Wiley: New York

Kaplan EL and Meier P (1958) Non-parametric estimation from incomplete observations. *J Am Stat Assoc* **53**: 457–481

Marty M, Pouillart P, Schol S, Droz JP, Azab M, Brion N, Pujade-Laraine E, Paule B, Paes D and Bons J (1990) Comparison of the 5-hydroxytryptamine (serotonin) antagonist ondansetron (GR38032F) with high-dose metoclopramide in the control of cisplatin-induced emesis. *N Engl J Med* **322**: 816–821

Mulder De PHM, Seynaeve C, Vermorken JB, Van Liessum PA, Mols-Jevdevic S, Allman EL, Beranek P and Verweij J (1990) Ondansetron compared with high-dose metoclopramide in prophylaxis of acute and delayed cisplatin-induced nausea and vomiting. *Ann Int Med* **113**: 834–840

Roila F, Tonato M, Cognetti F, Cortesi E, Favalli G, Marangolo M, Amadori D, Bella MA, Gramazio V, Donati D, Ballatori E and Del Favero A (1991) Prevention of cisplatin-induced emesis: a double-blind multicenter randomized crossover study comparing ondansetron and ondansetron plus dexamethasone. *J Clin Oncol* **9**: 675–678

Smith DB, Newlands ES, Rustin GJ, Begent RHJ, Howells N, McQuade B and Bagshawe KD (1991) Comparison of ondansetron and ondansetron plus dexamethasone as antiemetic prophylaxis during cisplatin-containing chemotherapy. *Lancet* **338**: 487–490

© Cancer Research Campaign 1998